# Personalized tumor-informed circulating tumor DNA monitoring for early detection of recurrence in postoperative pancreatic cancer

**DOI:** 10.3389/fonc.2026.1745466

**Published:** 2026-01-22

**Authors:** Jingjing Chen, Lu Zou, Xinyuan Bai, Fan Tong, Jiayao Ni, Haochen Tang, Yaru Liu, Xiang Kong, Jiani Yin, Fufeng Wang, Huizi Sha, Fanyan Meng, Juan Du

**Affiliations:** 1 Department of Oncology, Nanjing Drum Tower Hospital, Clinical College of Nanjing Drum Tower Hospital, Nanjing University of Chinese Medicine, Nanjing, China; 2 The Comprehensive Cancer Center of Nanjing Drum Tower Hospital, Affiliated Hospital of Medical School, Nanjing University, Nanjing, China; 3 Department of Oncology, Nanjing Drum Tower Hospital, Clinical College of Nanjing Medicine University, Nanjing, China; 4 Department of Oncology, Nanjing Drum Tower Hospital, Clinical Medical College of Jiangsu University, Zhenjiang, China; 5 Geneseeq Research Institute, Nanjing Geneseeq Technology Inc., Nanjing, China; 6 Department of Laboratory Medicine, Nanjing Drum Tower Hospital, Clinical College of Nanjing Medical University, Nanjing, China

**Keywords:** adjuvant chemotherapy, circulating tumor DNA, dynamic ctDNA, minimal residual disease, pancreatic cancer

## Abstract

**Background:**

Up to 80% of patients with resected pancreatic cancer experience recurrence within 2 years. We evaluated the feasibility and accuracy of a personalized, tumor-informed circulating tumor DNA (ctDNA) test for the early detection of recurrence risk during long-term postoperative surveillance.

**Methods:**

We recruited 43 patients with pancreatic cancer who underwent curative surgical resections. A personalized panel was developed to detect ctDNA in plasma based on whole-exome mutation information derived from tumor tissues. A total of 139 plasma samples were analyzed to assess recurrence risk and the efficacy of adjuvant therapy.

**Results:**

A personalized ctDNA monitoring panel was successfully customized in 35 of 43 cases. Sixteen patients relapsed within a median of 15.7 months (range: 5.4–30.0 months) postsurgery. For the 11 patients with positive ctDNA, the median lead time from initial ctDNA positivity to radiological relapse was 4.59 months (range: 0.88–15.61). After completion of adjuvant chemotherapy (ACT), 94.3% (33/35) of patients contributed 52.5% (73/139) of the ctDNA testing samples. These samples exhibited an elevated rate of ctDNA detection (48.5%, 16/33) compared to samples obtained prior to and during the commencement of ACT, with a negative predictive value of 82.4% (14/17) and a positive predictive value of 75.0% (12/16). The presence of ctDNA was significantly correlated with shorter disease-free survival and overall survival.

**Conclusions:**

Long-term dynamic ctDNA monitoring after pancreatic cancer resection, particularly following the completion of ACT, is predictive of recurrence risk. The proactive implementation of ctDNA monitoring after ACT in patients with resectable pancreatic cancer has important implications for clinical practice.

## Introduction

Pancreatic cancer is a highly aggressive gastrointestinal malignancy characterized by rapid invasion and poor prognosis, representing a major global public health challenge. It is projected to become the second leading cause of cancer-related mortality in the USA by 2030 (1). Only 10%–20% of patients are eligible for surgical resection (2). Despite radical pancreatectomy, approximately 90% of patients experience local recurrence or distant metastasis within 2 years, largely due to minimal residual disease (MRD) that remains undetected on imaging (3).

MRD is clinically occult and radiologically invisible at the time of surgery, and it is recognized as a major contributor to disease recurrence (4). Consequently, a standard treatment regimen comprising six to eight cycles of adjuvant chemotherapy (ACT) has been widely adopted, aiming to eliminate MRD.

While conventional serum cancer antigen detection and radiographic examination are commonly used for diagnosing or assessing the spread of pancreatic cancer, relying solely on these methods to identify patients with MRD is inadequate due to their limited sensitivity and specificity. Carbohydrate antigen 19-9 (CA19-9) lacks specificity, as its levels can also be elevated in benign conditions such as biliary inflammation or obstruction, complicating the differentiation of malignant disease (5–7). Furthermore, 5% to 10% of the general population do not express high levels of CA19–9 due to the absence of Lewis antigen expression (5–7). Conventional imaging modalities depend on the detection of macroscopically visible lesions (8) and are notably insensitive to identifying peritoneal metastases, making them unable to detect subclinical MRD.

Circulating tumor DNA (ctDNA) is defined as cell-free DNA released into the circulatory system by tumor cells undergoing apoptosis and necrosis. It has a short half-life (9), is highly tumor-specific, and can reflect dynamic changes in tumor burden in real time (10, 11). Noninvasive ctDNA-based surveillance has a broad range of applications in resectable pancreatic cancer. However, most studies have focused on a limited number of commonly mutated genes associated with pancreatic cancer, such as *KRAS* and tumor protein p53 (*TP53*) (12, 13), or have conducted ctDNA monitoring using fixed sequencing panels (14). A few studies have employed a personalized, tumor-informed approach (TIA) for ctDNA testing (15–17), which can enhance ctDNA detection rates in resectable pancreatic cancer (15).

Therefore, in this study, we employed ctDNA-based MRD monitoring to evaluate postoperative treatment outcomes and predict disease recurrence in patients with resectable pancreatic cancer. Our findings corroborate the high likelihood of recurrence associated with ctDNA positivity during the postoperative surveillance period, particularly after completion of adjuvant treatment.

## Materials and methods

### Study design and patient enrollment

This retrospective study was conducted at Nanjing Drum Tower Hospital, Affiliated Hospital of Medicine School, Nanjing University. The study protocol was reviewed and approved by the Nanjing Drum Tower Hospital Ethics Committee (2024-631-01) and complied with the ethical standards for medical research involving human subjects as outlined in the 1964 Declaration of Helsinki and its subsequent amendments. All participants provided written informed consent prior to enrollment. Patient treatment and follow-up were performed according to standard clinical practice, and data were extracted from the electronic medical record system.

Patients underwent multidisciplinary review and independent surgical oncological assessment to confirm resectability prior to enrollment. Individuals diagnosed with resectable pancreatic cancer who underwent radical pancreatic cancer resection between November 2021 and June 2023 and met the eligibility criteria were included in this study. The inclusion criteria were as follows: confirmed resectability, availability of complete clinical follow-up reports, and willingness to provide tumor tissue and ctDNA for mutation testing. Patients were excluded if their tumor tissue exhibited fewer than 16 monitorable single-nucleotide variant sites, as determined by whole-exome sequencing (WES), or if they were unable to comply with the treatment regimen. Consequently, 43 eligible patients were enrolled, and 35 patients were included in the survival analysis following successful customization of a personalized monitoring panel and ctDNA testing. The median postoperative follow-up period postsurgery was 15.69 months (range: 5.38–30 months).

### Adjuvant chemotherapy and follow-up

All patients received adjuvant treatment with follow-up in accordance with the Chinese Society of Clinical Oncology guidelines (18). ACT regimens included S-1/oxaliplatin/irinotecan (SOXIRI), modified fluorouracil/leucovorin/irinotecan/oxaliplatin (mFOLFIRINOX), and gemcitabine/tegafur–gimeracil–oteracil potassium (GS). Standard clinical follow-up included radiological assessments and serum cancer antigen tests for carcinoembryonic antigen (CEA), CA19-9, and carbohydrate antigen 125 (CA125), performed every 3 months during and after the treatment course until recurrence.

### Sample collection and DNA extraction

**Figure 1:**
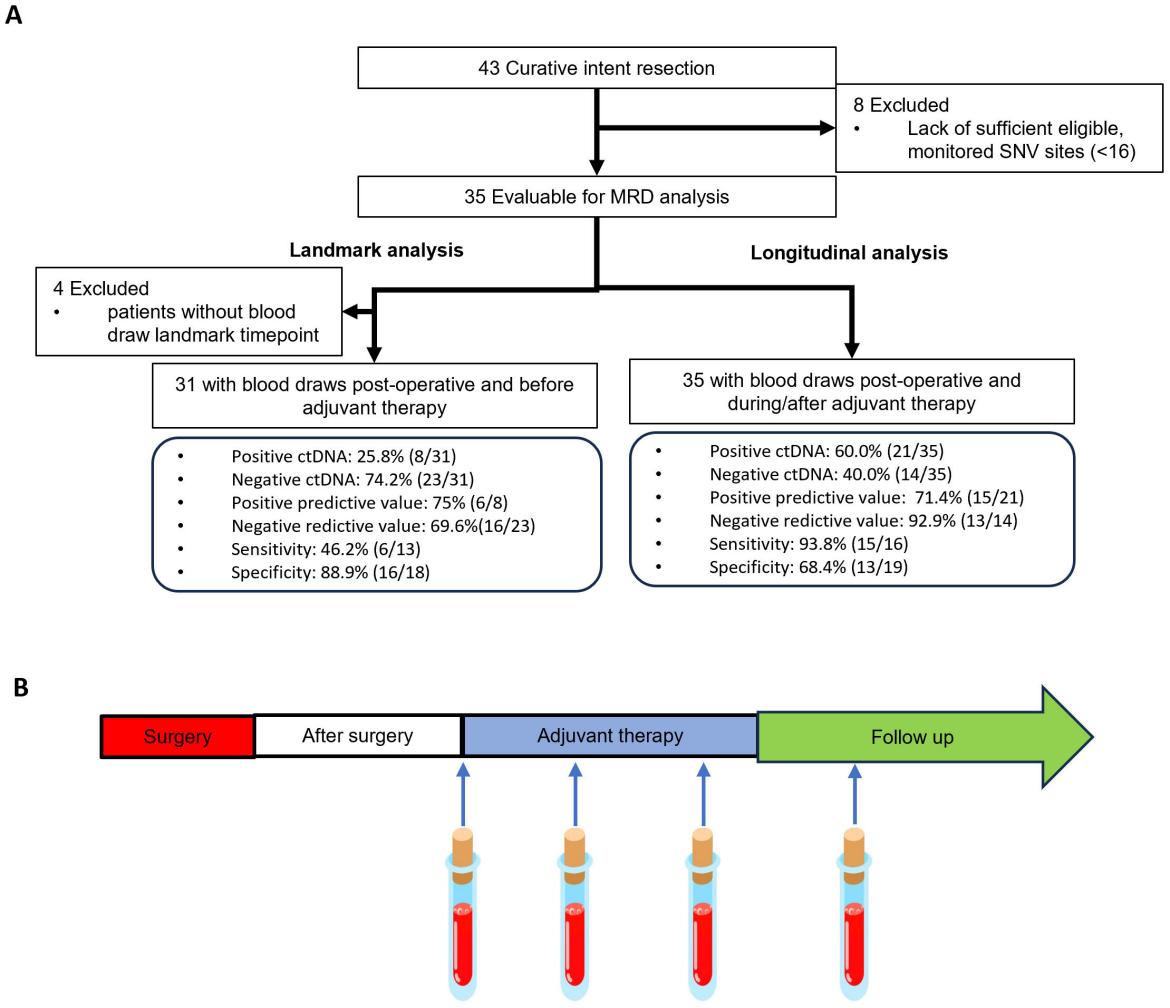
Schematics of patient enrollment and sample collection. **(A)** Patient inclusion for landmark analysis and longitudinal analysis for minimal residual disease (MRD) detection. **(B)** Plasma sample collection time points during the postoperative surveillance period. ctDNA, circulating tumor DNA.

The ctDNA analysis was performed on plasma samples collected postoperatively at a “landmark” time point prior to adjuvant therapy and at “longitudinal” time points throughout the postoperative surveillance period prior to recurrence (**Figure 1A**). The interval between each sampling time point was set at 3 months (**Figure 1B**). Ten milliliters of peripheral blood was collected at each sampling time point for subsequent ctDNA extraction.

Tumor DNA was extracted from formalin-fixed or surgically excised tumor tissues. Whole blood cells from peripheral blood DNA were used as a control for germline DNA analysis.

### Tumor-informed approach to ctDNA testing

A personalized, tumor-informed multiplex polymerase chain reaction (mPCR) next-generation sequencing (NGS) assay was employed for the detection and quantification of ctDNA, as previously described (19). Briefly, tumor-specific somatic single-nucleotide variants (SNVs) were identified through WES of tumor tissues (average sequencing depth: > 300 ×, with tumor-associated genes: > 500 ×) and matched germline DNA (average sequencing depth: > 100 ×), performed by OrigiMed in Shanghai, China. More than 16 clones were selected as custom loci for MRD panel sequencing (20). The detection limit for variant allele frequency (VAF) was 0.01%. A sample was defined as ctDNA-positive if two or more mutations specific to matched tumor tissues were detected in the plasma, indicating the presence of MRD.

### Statistical analyses

The primary outcome measures were disease-free survival (DFS) and overall survival (OS). DFS was calculated from the date of surgical resection to the date of confirmed radiological recurrence or death due to pancreatic cancer relapse. Radiological recurrence was assessed according to the RECIST guideline (version 1.1). OS was defined as the interval from the date of surgical resection to death or last follow-up, with the latter noted in cases of nontumor-related death. The association between ctDNA and clinical variables was evaluated using Cox proportional hazards regression analysis and Kaplan–Meier estimation. Results were reported with 95% confidence intervals (CI). All *p*-values were derived from two-sided tests, and differences were considered statistically significant at a *p*-value of less than 0.05.

## Results

### Patient clinicopathological characteristics

A total of 43 patients with stages I–III resectable tumors were enrolled in the study; eight were excluded due to having fewer than 16 monitorable clonal mutation sites in their tumor tissue. Consequently, 35 patients (81.4%) were successfully customized with a personalized MRD panel and included in the survival analysis. The median number of monitored SNV sites was 31 (range: 17–35). The clinicopathological characteristics of these 35 patients are summarized in **Table 1**. The pathological subtype of the resected tumor tissue was adenocarcinoma for most patients (*n* = 32, 91.4%). Nearly half of the patients (*n* = 17, 48.6%) were diagnosed at stage I, with 28.6% (*n* = 10) and 22.8% (*n* = 8) at stages II and III, respectively. The median age of the patients was 62.2 years (range: 41–74 years), and 25.7% (*n* = 9) had a history of tobacco use. All patients received more than one cycle of adjuvant chemotherapy, including SOXIRI (*n* = 19, 54.3%), mFOLFIRINOX (*n* = 8, 22.9%), and GS (*n* = 8, 22.9%).

**Table 1:** Clinical characteristics of the 35 pancreatic cancer patients enrolled for survival analysis.

Characteristics		Number	Ratio
Total		35	
Age	Mean 62.2 (range from 41-74) years		
Sex	Male	20	57.14%
	Female	15	42.86%
Stage	IA-IB	17	48.58%
	IIA-IIB	10	28.57%
	III	8	22.85%
ECOG PS	0	9	25.71%
	1	26	74.29%
R	R0	20	57.14%
	R1	14	40.00%
	R2	1	2.86%
Tumor location	Head/neck	24	68.57%
	Body/tail	11	31.43%
Tumor type	Adenocarcinoma	32	91.43%
	others	3	8.57%
Differentitation	Low	2	5.71%
	Middle	30	85.71%
	High	3	8.57%
Pathological tumor size	≥4cm	8	22.86%
	<4cm	27	77.14%
Pre-operative CA19-9 level	>37U/ml	24	68.57%
	≤37U/ml	11	31.43%
Pre-chemotherapy CA199 level	>37U/ml	7	20.00%
	≤37U/ml	27	77.14%
	unknown	1	2.86%
Surgical approach	PPPD	11	31.43%
	PD	11	31.43%
	TP	1	2.86%
	RAMPS	12	34.29%
Adjuvant therapy	mFOLFIRINOX	8	22.86%
	SOXIRI	19	54.28%
	GS	8	22.86%
patients experienced recurrence	stage I	5	31.25%
	stage II	6	37.50%
	stage III	5	31.25%
Experienced Disease Recurrence		**16**	**45.70%**
Death		**9**	**25.70%**
Days from Surgery to Recurrence – median (range)		**11.82 (2-17.77) months**	
Days of Clinical Follow Up from Surgery – median (range)		**15.69 (5.38-30) months**	

Bold values highlight key results.

**Figure 2:**
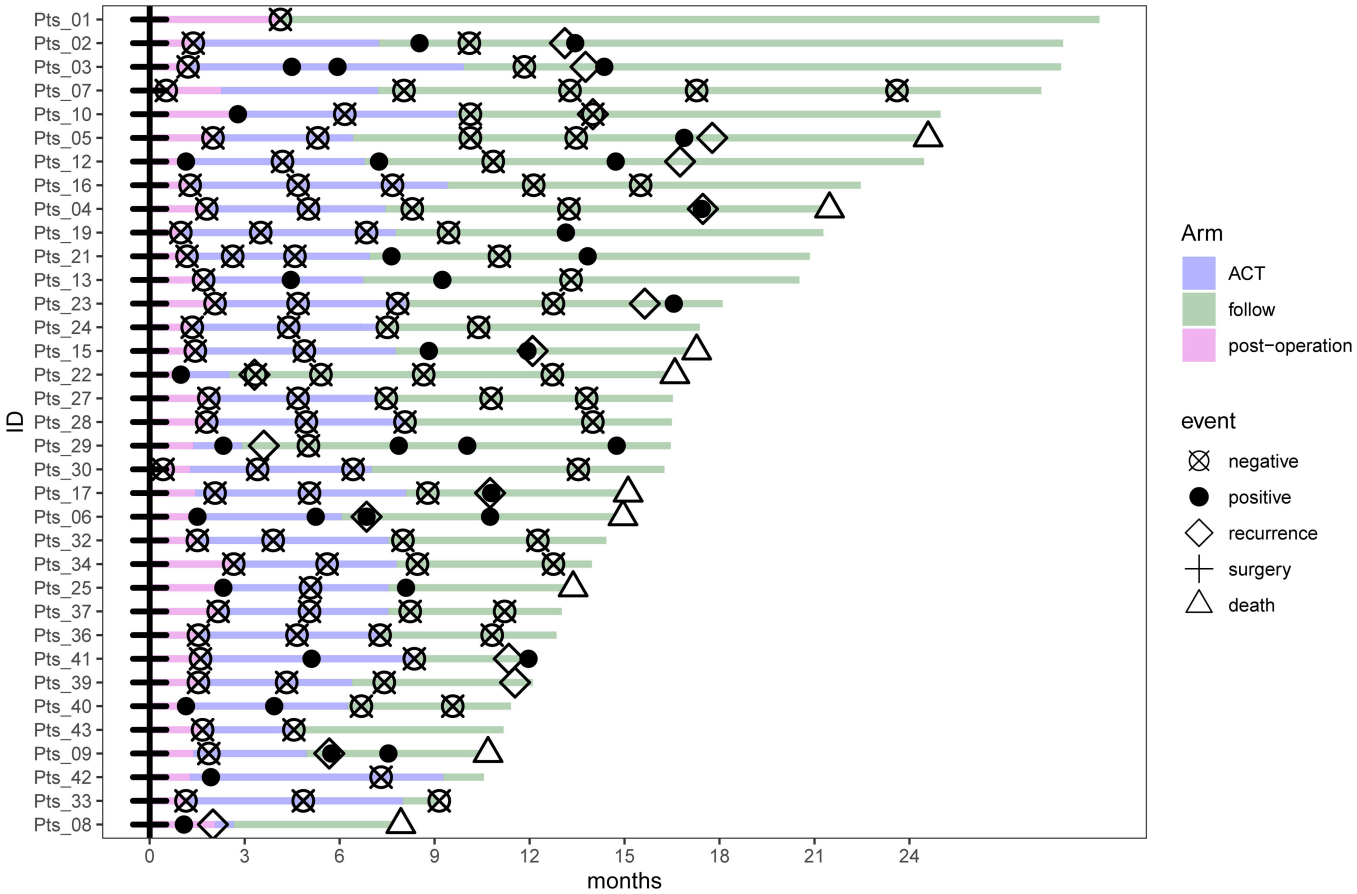
Swimmer plot depicting the minimal residual disease status (negative/positive) and tumor-related relapse or death events in patients during the postoperative surveillance period. ACT, adjuvant chemotherapy; follow, follow-up period.

A total of 139 plasma samples were collected from the 35 patients included in the survival analysis, with a median of four samples per patient (range: 1–6), to assess the clinical application of dynamic MRD monitoring. The timeline for surgery, adjuvant therapy, MRD positivity, recurrence, and death events for all patients is summarized in **Figure 2**. During a median postoperative follow-up of 15.69 months (range: 5.38–30 months), 16 patients (45.7%) experienced recurrence, and nine patients (25.7%) died. The median time to postoperative recurrence was 11.82 months (range: 2–17.77 months).

### Primary landmark analysis

**Figure 3:**
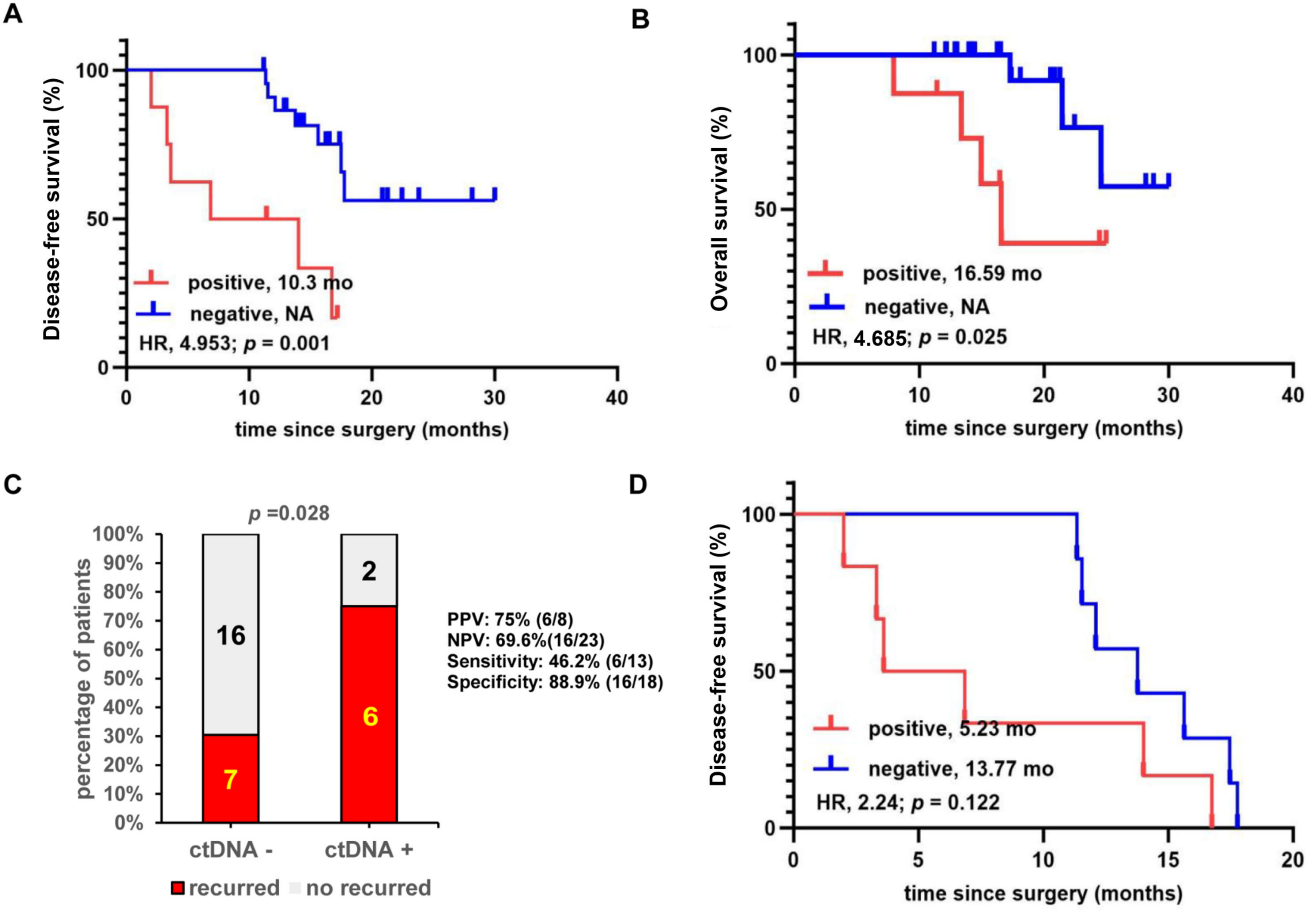
Landmark survival analysis for the association between patient prognosis and postoperative circulating tumor DNA (ctDNA) positivity (*N* = 31). **(A)** Kaplan–Meier comparison of disease-free survival between ctDNA-positive and ctDNA-negative patients. **(B)** Kaplan–Meier comparison of overall survival between ctDNA-positive and ctDNA-negative patients. **(C)** Comparison of recurrence rates between ctDNA-positive and ctDNA-negative patients. **(D)** Kaplan–Meier comparison of disease-free survival between ctDNA- and ctDNA-negative groups in the 16 relapsed patients. HR, hazard ratio; NA, not applicable; NPV, negative predictive value; PPV, positive predictive value.

A landmark plasma sample was collected for each patient before adjuvant therapy after radical surgery (**Figure 3**). Four patients were excluded from the landmark analysis due to a lack of landmark samples. The median time from surgery to plasma collection was 1.51 months (range: 0.43–4.13 months). This approach aimed to facilitate early MRD detection, which is critical for informing treatment decisions during the standard window for initiating adjuvant therapy.

For the 31 patients included in the primary landmark analysis, ctDNA was detectable in eight of 31 (25.8%) patients. Overall, patients with negative ctDNA at the landmark time point exhibited significantly longer median DFS(mDFS) (not available [NA] *vs*. 10.3 months; hazard ratio [HR]: 4.953; 95% CI: 1.069–22.94; *p =* 0.001) and median OS(mOS) (NA *vs*. 16.59 months; HR: 4.685; 95% CI: 0.7870 to 27.89; *p =* 0.025) than those with positive ctDNA (**Figures 3A, B**). ctDNA-positive patients also had a higher rate of recurrence (RR) of 75% (six of eight), compared to ctDNA-negative patients (RR = 30.4%, seven of 23, *p =* 0.028) (**Figure 3C**).

Of the eight ctDNA-positive patients, six experienced relapses (**Figures 1B**, **3C**; **Supplementary Table S1**) at a median of 5.23 months postoperatively, yielding a positive predictive value (PPV) for relapse of 75% (**Figure 3C**). Among the 23 patients in whom landmark ctDNA was not detected, seven experienced recurrences, with a median DFS of 13.77 months (range: 11.34–17.77 months). The negative predictive value (NPV) for relapse was 69.6% (16/23). The difference in mDFS between recurrent patients with and without ctDNA detected at landmark time points was more than twofold but did not reach statistical significance (13.77 months *vs*. 5.23 months; HR: 2.224; 95% CI: 0.661 to 7.482; *p =* 0.122; **Figure 3D**). The sensitivity and specificity of landmark ctDNA status for predicting recurrence were 46.2% (six of 13) and 88.9% (16/18), respectively. The ctDNA landmark test proved predictive of recurrence regardless of cancer stage (**Figure 3C**; **Supplementary Table S1**).

### Longitudinal ctDNA monitoring

**Figure 4:**
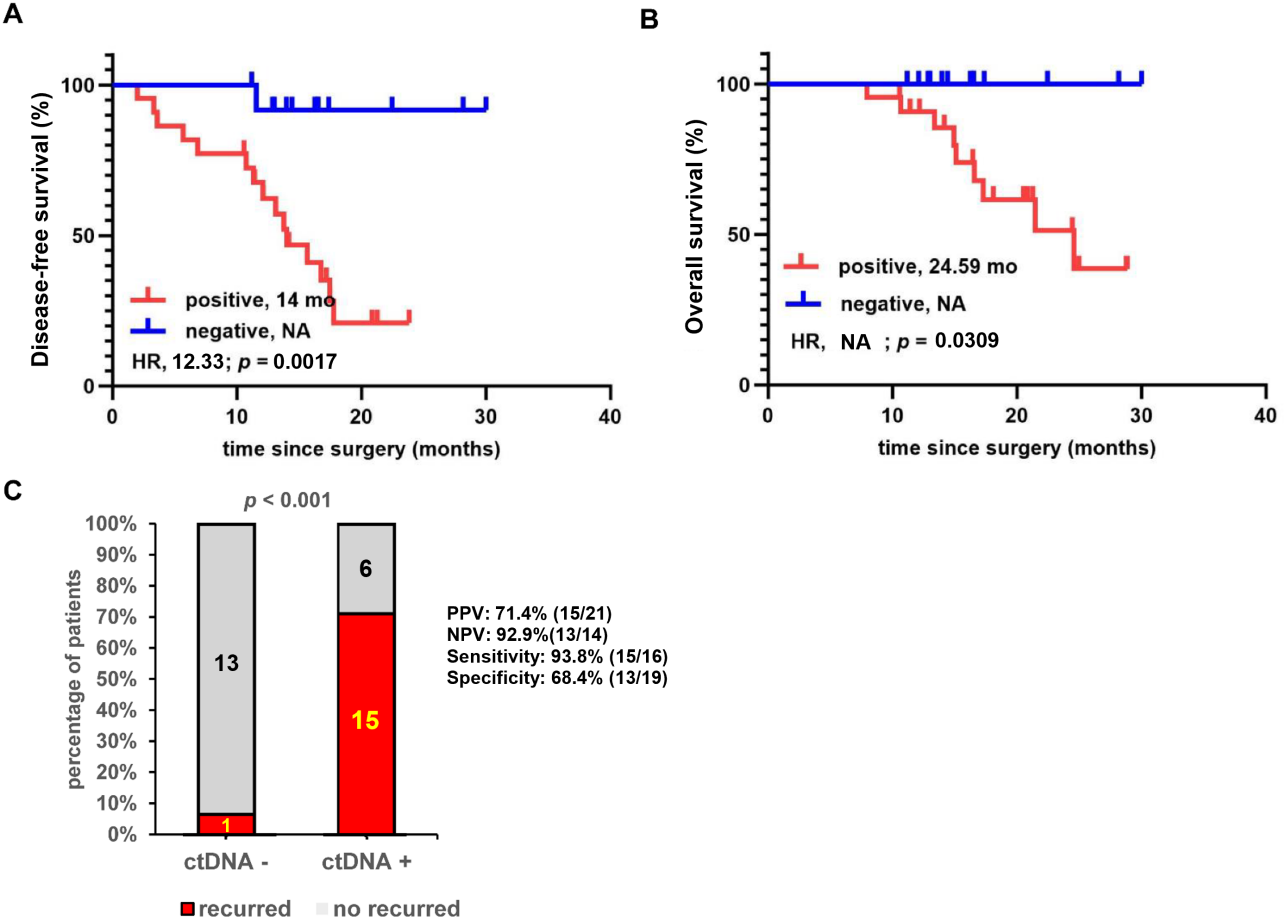
Longitudinal survival analysis focusing on the association between patient prognosis and circulating tumor DNA (ctDNA)–minimal residual disease (MRD) positivity throughout the monitoring process (*N* = 35). **(A)** Kaplan–Meier comparison of disease-free survival between ctDNA-MRD-positive and ctDNA-MRD-negative patients. **(B)** Kaplan–Meier comparison of overall survival between ctDNA-MRD-positive and ctDNA-MRD-negative patients. **(C)** Comparison of recurrence rates between ctDNA-MRD-positive and ctDNA-MRD-negative patients. HR, hazard ratio; NA, not applicable; NPV, negative predictive value; PPV, positive predictive value.

We performed a comprehensive analysis of ctDNA monitoring results throughout the postoperative period for all 35 patients (**Figure 4**). Of these, 21/35 (60.0%) were ctDNA-MRD-positive, and 14/35 (40.0%) were ctDNA-MRD-negative, with positive ctDNA in any of a patient’s plasma samples defined as ctDNA-MRD-positive. Compared to patients who were consistently ctDNA-negative, ctDNA-MRD-positive patients had significantly shorter mDFS (14 months *vs*. NA; HR: 12.33; 95% CI: 4.607 to 33.02; *p =* 0.0017) and mOS (24.59 months *vs*. NA; HR: NA; 95% CI: NA; *p =* 0.0309; **Figures 4A, B**).

Compared to ctDNA-negative patients, ctDNA-positive patients had a higher RR (ctDNA-positive: 71.4%, 15/21; ctDNA-negative: 7.1%, one of 14). At the last follow-up, 15 of the 21 ctDNA-MRD-positive patients experienced relapse, corresponding to a PPV for relapse of 71.4% (**Figures 1A**, **4C**). Of the 14 ctDNA-negative patients, only one experienced relapse, yielding a NPV of 92.9% (13/14). To evaluate long-term outcomes, additional follow-up was performed exclusively for the 14 ctDNA-negative patients. Using data updated as of 30 December 2025, three patients experienced relapse. The median DFS has not been reached, and the 2-year DFS rate is 78.6% (95% CI: 0.57 to 1.00). During the entire postoperative course, two patients provided ctDNA samples only once, both before adjuvant therapy (Pts_01: ctDNA not detected, no recurrence; Pts_08: ctDNA detected, recurred). Among the 11 of 16 (68.8%) patients with recurrence, the median lead time of ctDNA-MRD positivity prior to radiological progression was 4.59 months (range: 0.88–15.61) (**Supplementary Table S1**).

### Dynamic ctDNA-MRD change predicting patient relapse and survival

**Figure 5:**
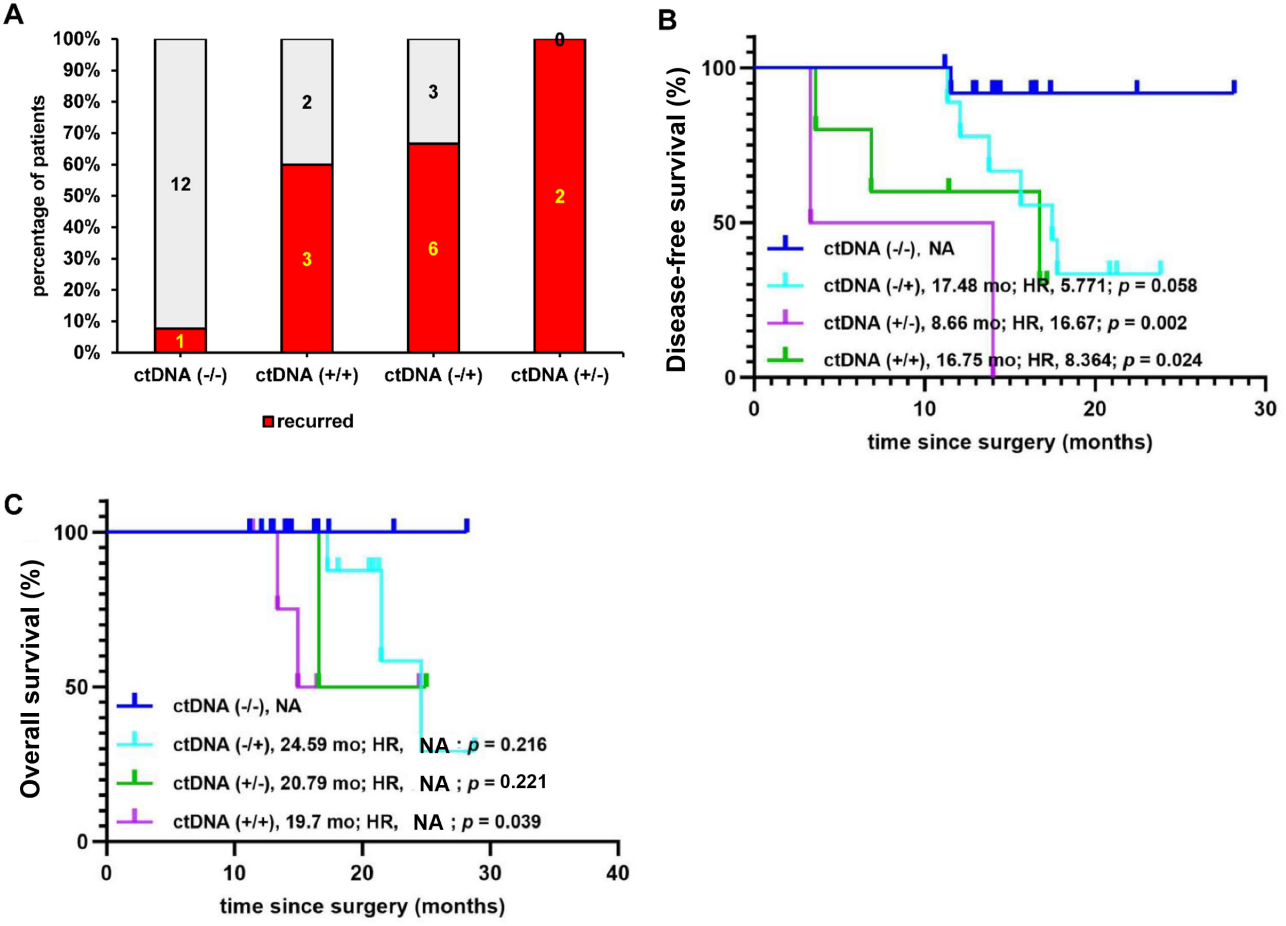
Dynamic change in circulating tumor DNA (ctDNA) positivity before and after adjuvant chemotherapy in relation to patient prognosis. **(A)** Comparison of recurrence rates for patients whose ctDNA remained negative (−/−), remained positive (+/+), switched from negative to positive (−/+), and switched from positive to negative (+/−). **(B)** Kaplan–Meier comparison of disease-free survival for the four patient groups. **(C)** Kaplan–Meier comparison of overall survival for the four patient groups. HR, hazard ratio; NA, not applicable.

We next investigated whether postoperative ctDNA dynamics correlated with relapse risk or survival probabilities. We compared the ctDNA status in paired samples collected before and after the start of adjuvant therapy. Among the 29 patients included in the analysis, 17.24% (five of 29) remained ctDNA-positive, 44.83% (13/29) remained ctDNA-negative, 31.03% (9/29) converted from negative to positive, and 6.9% (two of 29) converted from positive to negative (**Figure 5A**; **Supplementary Table S1**). When compared with the RR of patients whose ctDNA remained persistently negative (7.7%, one of 13), a higher RR was observed among patients who either remained persistently ctDNA-positive (RR: 60% [three of five]; Chi-squared *p =* 0.017) or converted from ctDNA-negative to ctDNA-positive (RR: 66.7% [six of nine]; Chi-squared *p =* 0.004), or converted from positive to negative (RR: 100% [two of two]; Chi-squared *p* = 0.002) (**Figure 5A**).

Further comparisons showed that patients who remained persistently ctDNA-negative demonstrated longer mDFS (NA *vs*. 16.75 months; HR: 8.364; 95% CI: 0.906 to 77.27; *p* = 0.024) and mOS (NA *vs*. 19.7 months; HR: NA; 95% CI: NA; *p =* 0.039) compared with those who remained persistently positive or converted from negative to positive (mDFS: NA *vs*. 17.48 months; HR: 5.771; 95% CI: 1.311 to 25.40; *p =* 0.058; mOS: NA *vs*. 24.59 months; HR: NA; 95% CI: NA; *p =* 0.216), or converted from positive to negative (mDFS: NA *vs*. 8.66 months; HR: 16.67; 95% CI: 0.429 to 647.2; *p =* 0.002; mOS: NA *vs*. 20.79 months; HR: NA; 95% CI: NA; *p =* 0.221) (**Figures 5B, C**).

### Comparing the predictive properties of ctDNA detected before and after ACT

**Figure 6:**
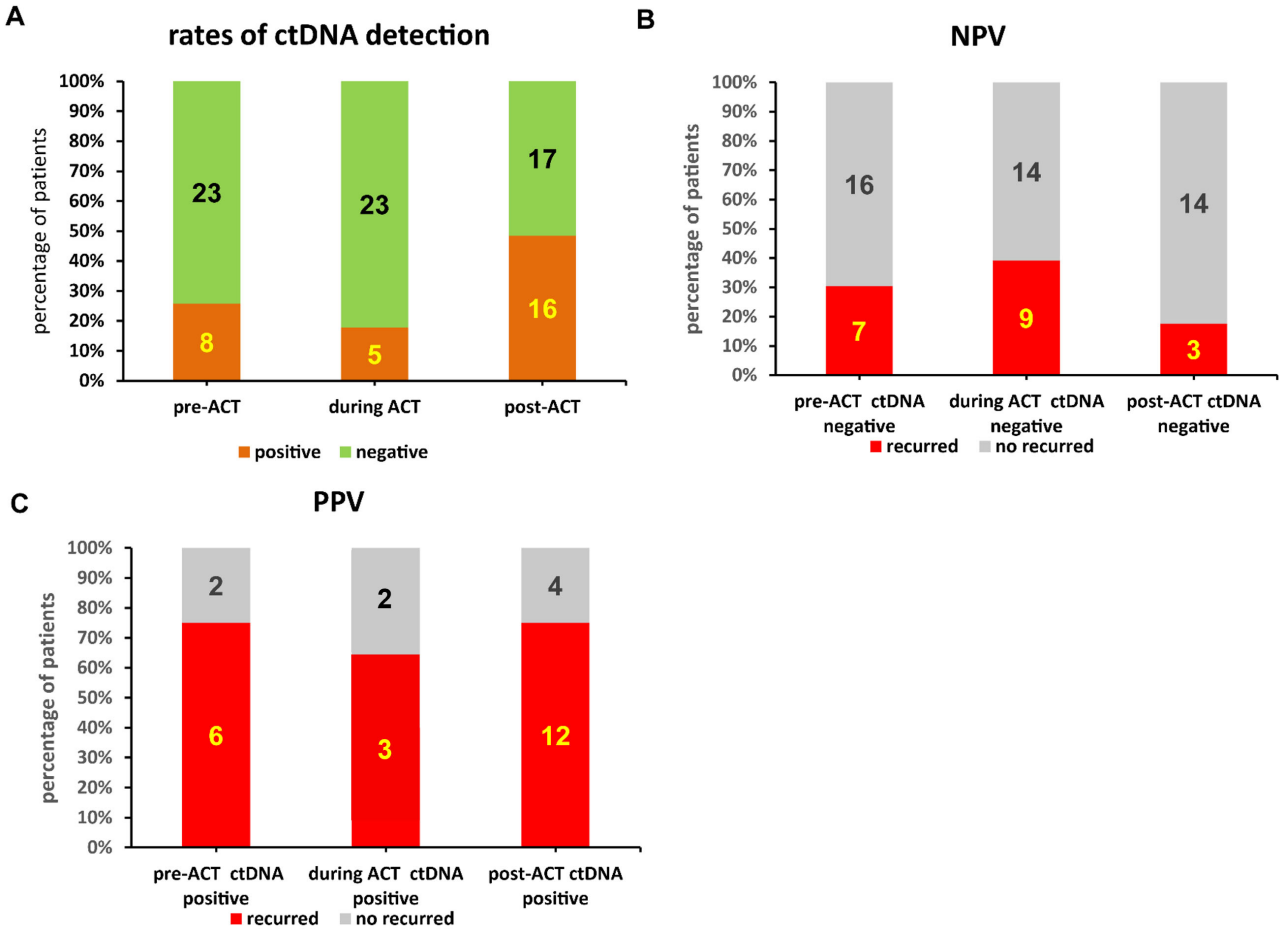
Comparisons of predictive capabilities of circulating tumor DNA (ctDNA) collected at different periods before, during, and after adjuvant chemotherapy (ACT). **(A)** Comparison of ctDNA detection rates before, during, and after ACT. **(B)** Comparison of negative predictive values (NPV) for relapse based on ctDNA positivity before, during, and after ACT. **(C)** Comparison of positive predictive values (PPV) for relapse based on ctDNA positivity before, during, and after ACT.

This study examined 139 ctDNA samples. Of these, 22.3% (31/139) were collected postoperatively and before ACT, covering 31/35 patients. A total of 25.2% (35/139) of the samples, covering 28/35 patients, were collected during ACT, and 52.5% (73/139), covering 33/35 patients, were collected after completion of ACT (**Figure 6A**). Compared to samples collected prior to ACT (ctDNA detection rate: 25.8%, eight of 31; NPV: 69.6%, 16/23) and during ACT (ctDNA detection rate: 17.9%, five of 28; NPV: 60.9%, 14/23), samples collected after ACT had the highest ctDNA detection rate (48.5%, 16/33) and NPV for relapse (82.4%, 14/17) (**Figures 6A, B**). The PPVs for ctDNA samples collected before (75%, six of eight) and after ACT (75%, 12/16) were also higher than those for samples collected during ACT (60%, three of five) (**Figure 6C**).

### Prognostic performance analysis of other factors

We also compared the effects of additional clinical markers and genomic features on prognostic performance, including stage, preoperative and postoperative CA19–9 levels, and mutations in the *KRAS* and *TP53* genes (**Supplementary Figure S1**). Patients with stage I pancreatic cancer had a better prognosis (mDFS: 17.77 months *vs*. 11.72 months; HR: 2.568; 95% CI: 0.722 to 9.131; *p =* 0.104; mOS: NA *vs*. 15.11 months; HR: 5.762; 95% CI: 1.338 to 24.82; *p =* 0.015) compared to those with stage II or stage III (mDFS: 17.77 months *vs*. 13.77 months; HR: 4.165; 95% CI: 0.868 to 20.00; *p =* 0.011; mOS: NA *vs*. NA; HR: 1.409; 95% CI: 0.107 to 18.48; *p =* 0.778) (**Supplementary Figures S1A, B**).

High preoperative CA19–9 levels (> 37 U/mL) showed no significant association with patient survival outcomes (mDFS: 17.48 months *vs*. NA; HR: 0.978; 95% CI: 0.338 to 2.828; *p =* 0.967; mOS: 24.59 months *vs*. NA; HR: 1.731; 95% CI: 0.432 to 6.939; *p =* 0.486) (**Supplementary Figures S1C, D**). In contrast, while high levels of postoperative CA19-9 (> 37 U/mL) did not predict recurrence (mDFS: 11.54 months *vs*. 17.77 months; HR: 2.430; 95% CI: 0.633 to 9.328; *p =* 0.087), they were associated with significantly shorter mOS (14.95 months *vs*. NA; HR: 5.721; 95% CI: 0.781 to 41.91; *p =* 0.003) (**Supplementary Figures S1E, F**). In our cohort, the presence of *KRAS* mutations did not affect mDFS (17.48 months *vs*. NA; HR: 2.097; 95% CI: 0.471 to 9.340; *p =* 0.463) and mOS (NA *vs*. NA; HR: 0.727; 95% CI: 0.068 to 7.732; *p =* 0.759) (**Supplementary Figures S1G, H**). Similarly, no differences in mDFS (15.64 months *vs*. 17.77 months; HR: 0.610; 95% CI: 0.165 to 2.254; *p =* 0.378) or mOS (24.59 months *vs*. NA; HR: 2.050; 95% CI: 0.405 to 10.37; *p =* 0.487) were observed when comparing patients with and without *TP53* mutations (**Supplementary Figures S1, J**).

## Discussion

Pancreatic cancer is characterized by high mortality rates and significant recurrence in surgical patients, making accurate early detection of recurrence critical. The AGITG DYNAMIC-Pancreas study, led by Lee et al., demonstrated the feasibility of using ctDNA to guide adjuvant chemotherapy duration and predict recurrence risk postsurgery in pancreatic cancer, independent of known prognostic markers (21). Most liquid biopsies for resectable pancreatic cancers focus on a single *KRAS* gene or a few commonly mutated genes, or they utilize sequencing panels encompassing a fixed set of tumor-related genes (12, 14, 22, 23). A limited number of studies have employed personalized TIA with known tumor-related genomic variations (16). It has been shown that targeting specific regions of individual genes results in limited mutation coverage and low detection rates for mutations in pancreatic cancer (24), which restricts ctDNA tests targeting fixed genomic regions. Conversely, TIA enables lower limit-of-detection tracking of identified mutation sites in tumors and is well-suited for liquid biopsies in pancreatic cancer. In this study, we adopted a detection strategy similar to the tumor-informed MRD product Signatera (25). We conducted WES testing on baseline tumor tissues to screen for somatic variants and designed individualized MRD detection panels for subsequent monitoring. The median number of monitored variants was 31 (range: 17–35), with a limit of detection (LOD) of 0.01%.

Our findings underscore the necessity of longitudinal ctDNA-MRD monitoring throughout the long-term clinical course of postoperative patients, particularly during and after ACT treatment. The ctDNA detection rate during the pre-ACT window was lower than that observed over the full monitoring period (25.8% *vs*. 62.9%), although the PPV for both periods was similar (75.0% *vs*. 68.2%). Notably, NPV and sensitivity improved from 69.6% (16/23) and 46.2% (6/13) to 92.9% (13/14) and 93.8% (15/16), respectively, when comparing the pre-ACT window with the full monitoring analysis. These combined results suggest that negative ctDNA results during the pre-ACT window are associated with a substantial proportion of false negatives, indicated by a high RR of 30.4% (seven of 23). In contrast, patients who remained negative throughout long-term monitoring exhibited a lower RR of 7.1% (one of 14). The post-ACT detection period showed the highest rate of ctDNA positivity, as well as the highest PPV and NPV for relapse, compared to previous detection periods. This suggests that ctDNA testing after completion of ACT possesses robust predictive capabilities, offering significant opportunities for early recurrence detection. Unlike the present study, previous personalized MRD studies in pancreatic cancer patients have not thoroughly evaluated the postoperative period following ACT (16, 26). Tumor-informed ctDNA-MRD monitoring is advancing toward a more comprehensive genomic scope, with strategies such as whole-genome sequencing (WGS) expected to enhance assay design, detection sensitivity, signal capture, and performance in low-mutation-burden tumors.

Limitations of this study include a limited sample size and variability in plasma collection time points. First, the relatively small patient cohort limits the ability to conduct a comprehensive and detailed analysis. In this study cohort, a subset of patients could not develop individualized tumor-informed detection panels due to an insufficient number of detectable tumor-specific mutations. Consequently, survival and predictive analyses were restricted to patients for whom the panels were successfully established, potentially introducing selection bias and possibly overestimating the assay’s feasibility and sensitivity in the general population. For these patients, tumor-naive MRD detection may serve as an alternative option, though further clinical validation is warranted. The high proportion of post-ACT time periods within the full monitoring period underscores the necessity for long-term MRD monitoring both before and after ACT. Additionally, the variability in plasma sampling time points reflects a common challenge in clinical practice, particularly in developing countries. Economic constraints, limited awareness of the significance of ctDNA monitoring, and limited access to healthcare resources may prevent patients from receiving consistent and timely ctDNA assessments. Our study underscores the importance of monitoring both before and after ACT and provides valuable data to optimize healthcare investment, highlighting the implications for actionable clinical practices.

In conclusion, a positive postoperative ctDNA status in pancreatic cancer patients was significantly associated with a higher risk of recurrence and poorer survival. Comprehensive dynamic monitoring of ctDNA can inform clinical decision-making, and future large-scale clinical trials are expected to validate these findings.

## Data Availability

The original contributions presented in the study are included in the article/**Supplementary Material**. Further inquiries can be directed to the corresponding authors.

## Supplementary material

Supplementary Figure 1 Kaplan-Meier comparisons of survival probabilities for patients with different clinical or mutational features. **(A)** Comparison of disease-free survival (DFS) among patients of different stages. **(B)** Comparison of overall survival (OS) among patients of different stages. **(C)** Comparison of DFS between patients of high (>37U/ml) and low (<37U/ml) preoperative CA19–9 levels. **(D)** Comparison of OS between patients of high (>37U/ml) and low (<37U/ml) preoperative CA19–9 levels. **(E)** Comparison of DFS between patients of high (>37U/ml) and low (<37U/ml) postoperative CA19–9 levels. **(F)** Comparison of OS between patients of high (>37U/ml) and low (<37U/ml) postoperative CA19–9 levels. **(G)** Comparison of DFS between patients with (*KRAS*-mutated) and without (*KRAS*-wild) *KRAS* mutations. **(H)** Comparison of OS between patients with (*KRAS*-mutated) and without (*KRAS*-wild) *KRAS* mutations. **(I)** Comparison of DFS between patients with (*TP53*-mutated) and without (*TP53*-wild) *TP53* mutations. **(J)** Comparison of OS between patients with (*TP53*-mutated) and without (*TP53*-wild) *TP53* mutations.

Supplementary Table 1 Individual testing and survival information of the 35 pancreatic cancer patients enrolled for survival analysis. ctDNA, circulating tumor DNA; DFS, disease-free survival; OS, overall survival.
