# Supplementary figures and images for: Personalized tumor-informed circulating tumor DNA monitoring for early detection of recurrence in postoperative pancreatic cancer
Source: Front Oncol. 2026 Jan 22;16:1745466. doi: 10.3389/fonc.2026.1745466 (PMC12872575; doi:10.3389/fonc.2026.1745466)

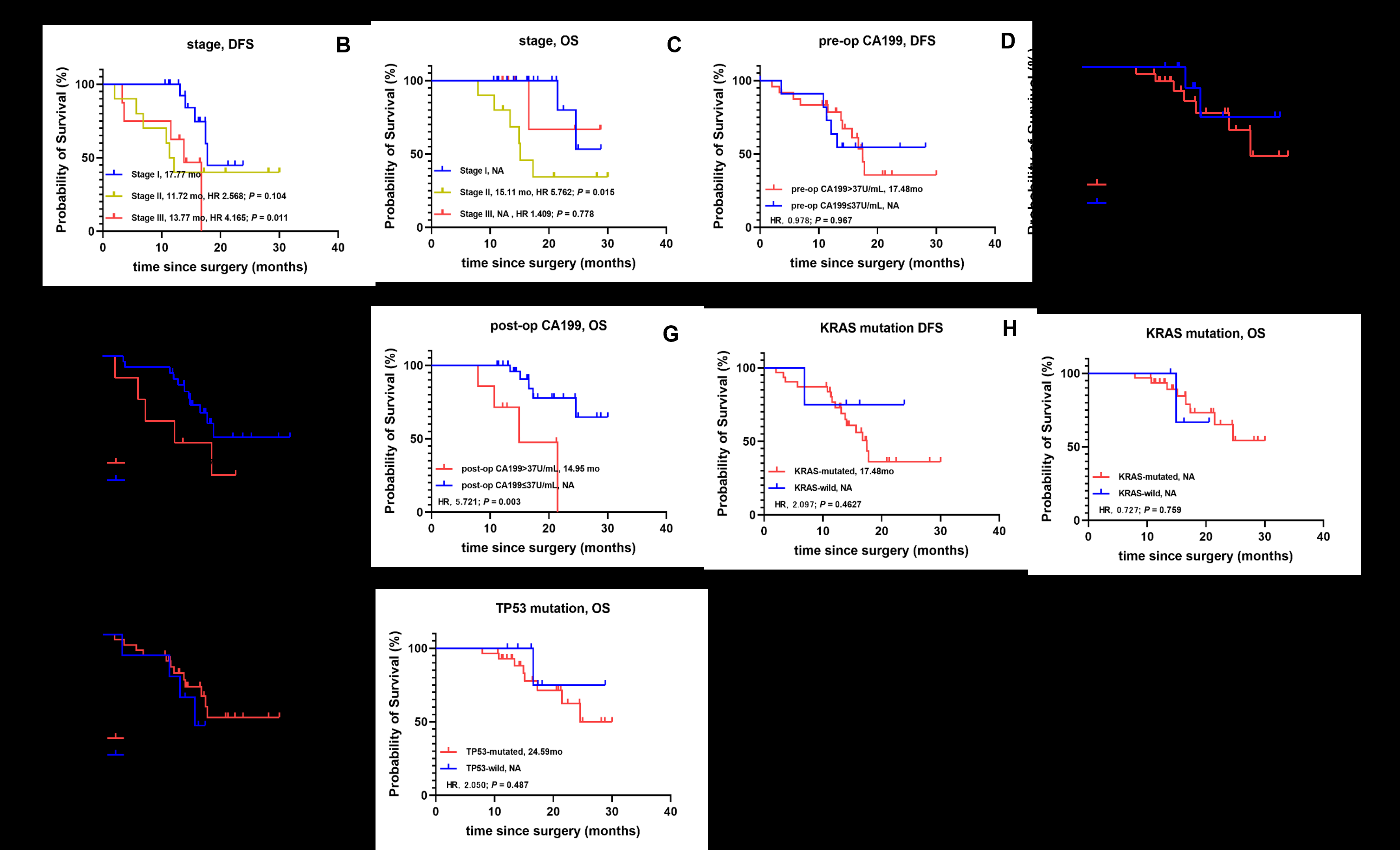

Supplement: Supplementary Figure 1 — Kaplan-Meier comparisons of survival probabilities for patients with different clinical or mutational features. (A) Comparison of disease-free survival (DFS) among patients of different stages. (B) Comparison of overall survival (OS) among patients of different stages. (C) Comparison of DFS between patients of high (>37U/ml) and low (<37U/ml) preoperative CA19–9 levels. (D) Comparison of OS between patients of high (>37U/ml) and low (<37U/ml) preoperative CA19–9 levels. (E) Comparison of DFS between patients of high (>37U/ml) and low (<37U/ml) postoperative CA19–9 levels. (F) Comparison of OS between patients of high (>37U/ml) and low (<37U/ml) postoperative CA19–9 levels. (G) Comparison of DFS between patients with (KRAS-mutated) and without (KRAS-wild) KRAS mutations. (H) Comparison of OS between patients with (KRAS-mutated) and without (KRAS-wild) KRAS mutations. (I) Comparison of DFS between patients with (TP53-mutated) and without (TP53-wild) TP53 mutations. (J) Comparison of OS between patients with (TP53-mutated) and without (TP53-wild) TP53 mutations. [file Image1.tif]
